# The Choice of a Medical School

**Published:** 1898-09-03

**Authors:** 


### The Choice of a Medical School.

Among all the numerous medical schools which exist in this country we doubt if there is one in which it would be impossible for an intelligent young fellow to pick up a good, sound, useful medical education. It is only fair, then, to allow other considerations, besides those which bear upon the comparative facilities for study offered by the various schools, to have weight in deciding where to go to train for one's profession. The course of study which is now required is so long that the expense of a student's mere keep is a matter of no small moment, and in many cases the fact that there is a medical school in a man's own town or within convenient distance, or the possibility of boarding with friends or relatives in some particular town where a school exists, may very properly be a deciding influence in the choice of a school, while the possession of friends or relatives on the staff of a particular hospital may also drive a youth to one place rather than to another. Again, the question of economy is often a very urgent one, and a comparatively small saving in expense, either in fees or "keep," may turn the scale with many. Putting all such considerations, however, on one side, we have to consider the questions of large schools or small, large hospitals or small ones, schools in connection with colleges or connected merely with hospitals, and the great question of schools which are directly connected with universities, as against those which have no other such connexion than that which all schools have with the University of London.

Large Schools or Small.

We have no doubt that for a clever youth a large school is the best; the classes are more subdivided, the teaching appliances are generally better, and very often the teaching itself is better also. Not that the teachers are more skilful surgeons or physicians, but at a large school it is more worth while for the best men to devote a large amount of trouble to mere teaching than it is where the numbers are but small. Moreover, the greater the number of the students the greater is the likelihood of there being among them some of those exceptionally brilliant men whose presence in a class forces a teacher to put forth his best powers. A leaven of such men is not always to be found in a small school, yet their presence is of infinite advantage, for the character of the teaching which is given in a school often depends almost as much upon the demand as on the supply, on the taught almost as much as on the teacher.

Again, a clever youth does best where he is sure to rub against others cleverer than himself, and nothing can be worse for him than to be *facile princeps* in a little school. Nor must monetary considerations be put out of sight. A large school is generally much better supplied with prizes and exhibitions than a small one, and all these things go into the pockets of the clever students.

On the other hand, the backward student often loses much of the advantage which a large school offers to his cleverer companion; he is not stimulating to the teacher, who, finding plenty of better material to occupy his time, leaves the backward one alone, to become more backward still. We think that for the shy, the backward, and the diffident a small school is the best—a school in which the teachers are more in personal and friendly touch with the students than is possible in larger institutions.

Small Hospitals or Large.

Again the large hospital has a great pull over the small one, more particularly in regard to the greater completeness of the special departments. But in regard to size of hospital this should be remembered, that unless the school is large in proportion a great deal of work is apt to be put upon the students, more, in fact, than they can properly do.

It is better that a large school should be worked in connection with a small hospital than that a small school should have the run of a very large hospital, for in the latter case slovenly work is almost sure to creep in.

Colleges.

Theoretically, it should be a great advantage to all parties that a medical school should be but one department in a college where many subjects are taught. This is the case at King's College; University College; Mason College, Birmingham; the Owens College, Manchester; the Yorkshire College, Leeds; and others. And, of course, at Cambridge the medical are mixed up with other students in the various colleges. This ought to favour a wider culture and to give a broader tone.

Unfortunately, in practice medical students tend to be gregarious; and even at Cambridge, admirable as is the school, it may be doubted whether the medical element does not follow its own tendencies and separate itself to a large extent from those who, working at very different subjects, soon develop different interests.

We do not think, much as has been made of this point, that the connection of a medical school with a college for general teaching is a matter of the slightest importance one way or the other.

Universities.

The connection of a medical school with a university is, however, quite another matter. We can have no possible doubt of the great benefit that it is to a student to belong to a school which is directly connected with the university from which he will look to receive his degree, and we hope that now at last, through the action of the Commission appointed by the London University Act, the medical schools of London will find themselves possessed of at least some of the advantages which the medical faculties of other universities have long enjoyed.

London or Provincial.

Finally, we come to the question which is usually the first to be asked, Should a youth be sent to a London or a provincial school? Now, in regard to this, it may at once be said that some of the provincial schools, considered as teaching institutions, are quite equal to many London ones and superior to some, so that if it were merely a question where best to obtain a medical education, there would be but little to choose between them. Where, then, it is certain that the student's work in life will lie in general practice, or in the colonies, or in the army, and especially where his interests tend to attach him permanently to any particular provincial school, the provinces will do as well as, and in some cases better than, London. We cannot, however, shut our eyes to the fact that the highest prizes of the profession fall into the hands of those who make themselves a name in London, and that a provincial education is not an open sesame to the London hospitals. However good a provincial education may be as an opening to a provincial career, we think that a London education, a London diploma, and a good place in the lists at the London University, followed, if possible, by a period of study in Germany, form the best introduction to London hospital practice, and thus to the highest prizes of the profession.

